# Blue Light Induces Impaired Autophagy through Nucleotide-Binding Oligomerization Domain 2 Activation on the Mouse Ocular Surface

**DOI:** 10.3390/ijms22042015

**Published:** 2021-02-18

**Authors:** Ying Li, Rujun Jin, Lan Li, Ji Suk Choi, Jonghwa Kim, Hyeon Jeong Yoon, Jong Hwan Park, Kyung Chul Yoon

**Affiliations:** 1 Department of Ophthalmology, Chonnam National University Medical School and Hospital, Gwangju 61469, Korea; leeyeong9989@gmail.com (Y.L.); rujun0611@daum.net (R.J.); lilan1207@naver.com (L.L.); kkangirri@hanmail.net (J.S.C.); ccaaacc@daum.net (J.K.); yoonhyeonjeong@hanmail.net (H.J.Y.); 2 Department of Biomedical Sciences and Centers for Creative Biomedical Scientists, Chonnam National University, Gwangju 61469, Korea; 3 Laboratory of Animal Medicine, College of Veterinary Medicine and BK21 Plus Project Team, Chonnam National University, Gwangju 77, Korea; jonpark@jnu.ac.kr

**Keywords:** blue light, nucleotide-binding oligomerization domain 2, autophagy related 16 like 1, autophagy, apoptosis

## Abstract

In this study, we investigated the effects of blue light exposure on nucleotide-binding oligomerization domain 2 (NOD2) expression on the mouse ocular surface and evaluated the role of NOD2 activation in light-induced cell death. Mice were divided into wild-type (WT), NOD2-knock out (KO), WT + blue light (WT + BL), and NOD2-KO + blue light (NOD2-KO + BL) groups, and the mice in the WT+BL and NOD2-KO + BL groups were exposed to blue light for 10 days. After 10 days of blue light exposure, increased reactive oxygen species and malondialdehyde were observed in the WT + BL and NOD2-KO + BL groups, and the WT + BL group showed a higher expression of NOD2 and autophagy related 16 like 1. Although both WT+BL and NOD2-KO + BL groups showed an increase in the expression of light chain 3-II, NOD2-KO + BL mice had a significantly lower p62 expression than WT + BL mice. In addition, NOD2-KO+BL mice had significantly lower corneal epithelial damage and apoptosis than WT + BL mice. In conclusion, blue light exposure can induce impaired autophagy by activation of NOD2 on the ocular surface. In addition, the reactive oxygen species (ROS)–NOD2–autophagy related 16 like 1 (ATG16L) signaling pathway may be involved in the blue-light-induced autophagy responses, resulting in corneal epithelial apoptosis.

## 1. Introduction

Humans are constantly exposed to various types of light, and light-induced ocular pathologies have been recognized, including photokeratitis, pterygium, climatic droplet keratopathy, cataracts, and corneal and retinal degeneration [1,2,3,4]. Recently, studies have shown that blue light causes photoreceptor and retinal pigment epithelial (RPE) cell damage by inducing excessive reactive oxygen species (ROS) production [5,6]. Our previous study demonstrated that blue light exposure can induce apoptosis in the ocular surface of mice by increasing oxidative stress and inflammatory molecules, thus aggravating clinical dry eye parameters, including tear volume, tear film break-up time, and corneal epithelial staining scores [7].

Autophagy is a conserved process that recycles defective cellular organelles and macromolecules. The process is upregulated in conditions such as oxidative stress, when cells need to remove unnecessary or dysfunctional components [8]. Recently, several studies have shown that blue light irradiation promotes apoptosis by the induction of impaired autophagy in RPE cells [6].

Pattern recognition receptors such as toll-like receptors (TLRs) and nucleotide binding and oligomerization domain (NOD)-like receptors (NLRs) mediate the innate immune response receptors by detecting imminent dangers [9,10]. Several studies have shown that short-wavelength light, including blue light, can induce NLRP 3 inflammasome activation in RPE cells [5,11].

Nucleotide-binding oligomerization domain 2 (NOD2), an NLR, detects characteristic microbial products and danger signals [12,13,14]. We have reported that NOD2 signaling is involved in the pathogenesis of dry eye and aggravates clinical and experimental parameters [15,16]. Interestingly, increasing evidence has shown that NOD2 stimulation impairs autophagy response in dendritic cells and that increased activation of autophagy related 16 like 1 (ATG16L1) signaling upregulates the induction of deficient autophagy in Crohn’s disease [13,14,17,18]. In addition, a previous study has reported that reactive oxygen species (ROS)-mediated NOD2 activation further impairs epithelial barrier function in inflammatory bowel disease (IBD) [18].

Although the role of NLRP 3 inflammasome after exposure to blue light has been investigated in the retina, the expression pattern of NOD2 on the ocular surface after blue light exposure has not been investigated. In the present study, we investigated the effects of blue light exposure on NOD2 activation on the ocular surface, and further explored the role of NOD2 activation in blue-light-induced cell death in both wild-type (WT) and NOD2-knockout (KO) C57/BL6 mice.

## 2. Results

### 2.1. ROS Levels on the Ocular Surface

**Figure 1:**
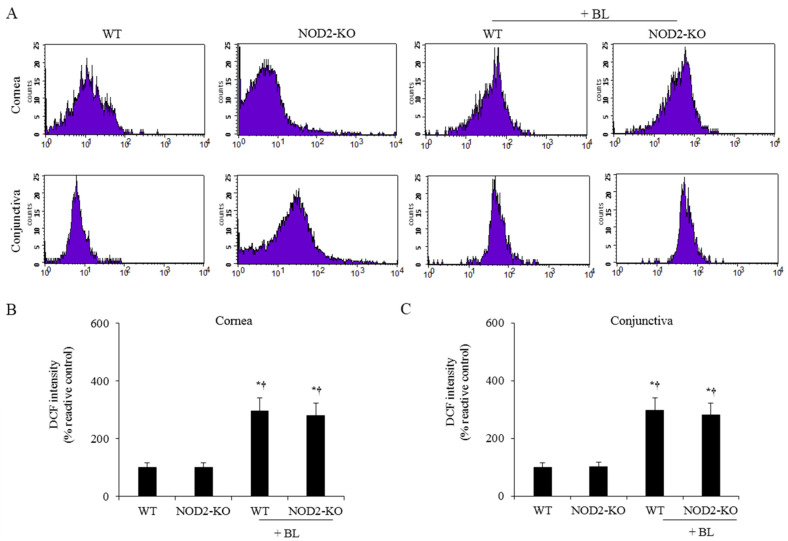
2′,7′-Dichlorodihydrofluorescein diacetate (DCF-DA) assay of representative photograph (**A**) and the mean of staining in cornea (**B**) and conjunctiva (**C**) of the WT, NOD2-KO, WT+BL, and NOD2-KO+BL groups, after 10 days of blue light exposure. * *p* < 0.05 *vs.* WT; ^†^ *p* < 0.05 *vs.* NOD2-KO. WT, wild type; BL, blue light; NOD2-KO, nucleotide-binding oligomerization domain 2 knock-out.

To investigate ROS caused by blue light exposure, we performed 2′,7′-dichlorodihydrofluorescein diacetate (DCF-DA) assays in the cornea and conjunctiva (Figure 1). Both WT + BL and NOD2-KO + BL mice showed significantly higher levels of ROS in the cornea and conjunctiva compared to the WT (all *p* < 0.01) and NOD2-KO mice (all *p* < 0.01).

### 2.2. MDA Levels on the Ocular Surface

After 10 days of blue light exposure, both WT+BL and NOD2-KO+BL mice showed significantly higher levels of malondialdehyde (MDA) in the cornea and conjunctiva than WT (all *p* < 0.01) and NOD2-KO mice (all *p* < 0.01). In the control groups, there were no significant differences between WT and NOD2-KO mice (Table 1).

### 2.3. NOD2 Activation in Corneal and Conjunctival Tissues

**Figure 2:**
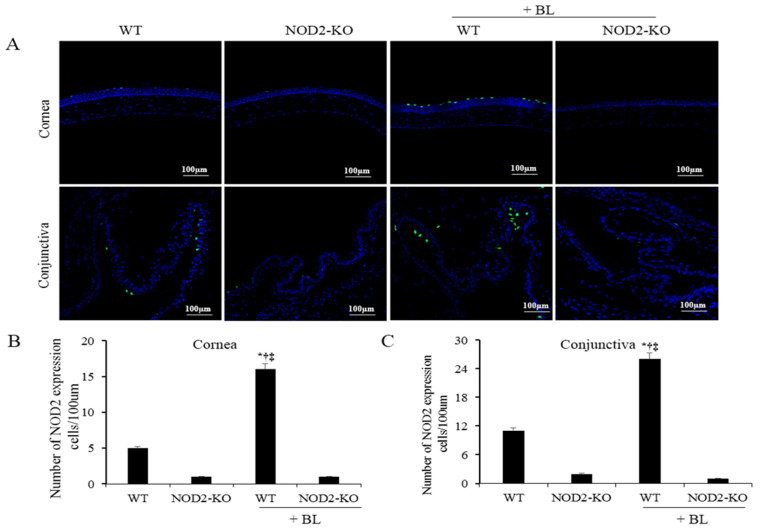
Immunofluorescent staining of representative photograph (**A**) and the mean number of NOD2 expression in corneal (**B**) and conjunctival (**C**) tissues of the WT, NOD2-KO, WT+BL, and NOD2-KO+BL groups, after 10 days of blue light exposure. * *p* < 0.05 *vs.* WT; ^†^ *p* < 0.05 *vs.* NOD2-KO ‡ *p* < 0.05 *vs.* WT + BL; WT, wild type; BL, blue light; NOD2-KO, nucleotide-binding oligomerization domain 2 knock-out.

Magnified images of the representative corneal sections stained with NOD2 (green) and counterstained with 4′,6-diamidino-2-phenylindole (DAPI) (blue) are shown in Figure 2A. The mean NOD2-positive cell counts in the corneal epithelium at 10 days were 4.3 ± 2.2 cells/100 μm in the WT, 0.8 ± 0.5 cells/100 μm in the NOD2-KO, 18.3 ± 7.3 cells/100 μm in the WT+BL, and 0.5 ± 0.8 cells/100 μm in the NOD2-KO+BL, respectively (Figure 2B). The mean NOD2-positive cell counts in the conjunctiva were 6.8 ± 1.3 cells/100 μm, 1.0 ± 0.8 cells/100 μm, 24.2 ± 2.6 cells/100 μm, and 1.1 ± 0.8 cells/100 μm in the WT, NOD2-KO, WT+BL, and NOD2-KO+BL groups, respectively (Figure 2C). Mice in the WT+BL group had a significantly higher expression of NOD2 in the cornea and conjunctiva than in the WT (*p* < 0.01), NOD2-KO (*p* < 0.01), and NOD2-KO+BL groups (*p* < 0.01).

### 2.4. Western Blot for Autophagy in Corneal and Conjunctival Tissues

**Figure 3:**
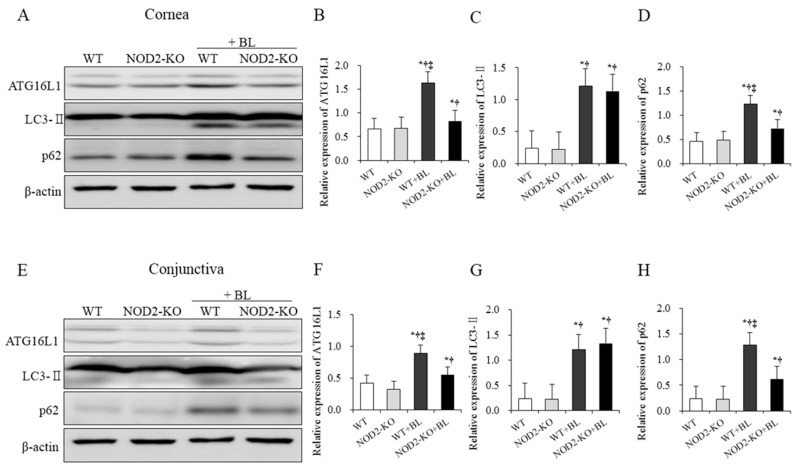
Western blot analysis of autophagy related 16 like 1 (ATG16L1), light chain 3-II (LC3-II), and p62 expression in the WT, NOD2-KO, WT+BL, and NOD2-KO+BL groups after 10 days of blue light exposure. Representative immunoblots of cell lysates showing detection of phosphorylated ATG16L1, LC3-II, and p62 with β-actin loading control in the cornea (**A**) and conjunctiva (**E**). Relative protein expression of ATG16L1, LC3-II, and p62 in the cornea (**B**–**D**) and conjunctiva (**F**–**H**). * *p* < 0.05 *vs.* WT; ^†^ *p* < 0.05 *vs*. NOD2-KO; ‡ *p* < 0.05 vs. WT + BL. WT, wild type; BL, blue light; NOD2-KO, nucleotide-binding oligomerization domain 2 knock-out.

A significantly higher expression of ATG16L1 and p62 were noted in the WT+BL group than in the WT, NOD2-KO, and NOD2-KO+BL groups (all *p* < 0.01). In addition, mice in the NOD2-KO+BL group had a significantly lower p62 expression in the cornea and conjunctiva than those in the WT+BL group (all *p* < 0.05). The WT+BL and NOD2-KO+BL groups showed higher expression of light chain 3-II (LC3-II) compared to the WT and NOD2-KO control groups (all *p* < 0.05). No significant differences were found between WT and NOD2-KO control groups (Figure 3).

### 2.5. Counts of Apoptotic Cells in Corneal Tissue

**Figure 4:**
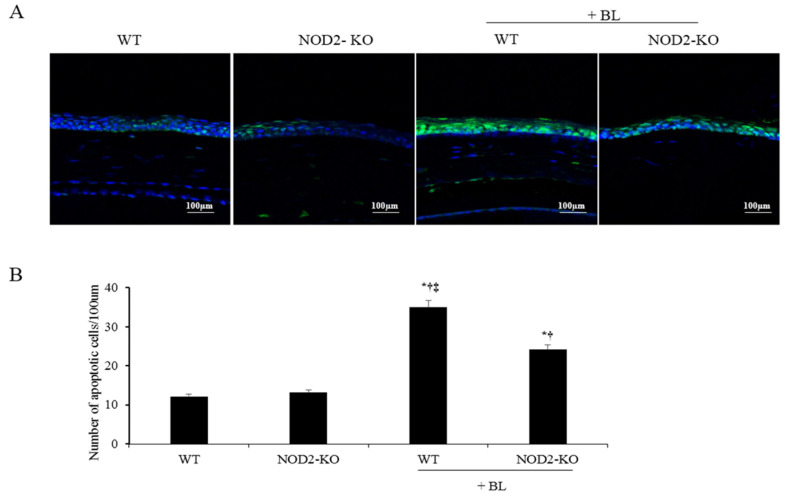
Terminal deoxynucleotidyl transferase-mediated dUTP-nick end labeling (TUNEL) staining of representative photograph (**A**) and the mean number of TUNEL-positive cells (**B**) in the cornea of the WT, NOD2-KO, WT+BL, and NOD2-KO+BL groups, after 10 days of blue light exposure. * *p* < 0.05 *vs.* WT; ^†^ *p* < 0.05 *vs.* NOD2-KO; ‡ *p* < 0.05 vs. WT + BL. WT, wild type; BL, blue light; NOD2-KO, nucleotide-binding oligomerization domain 2 knock-out.

Magnified images of the representative corneal sections stained with terminal deoxynucleotidyl transferase-mediated dUTP-nick end labeling (TUNEL) (green), and counterstained with DAPI (blue), are shown in Figure 4A. The mean apoptotic cell counts in the cornea were 11.5 ± 2.8 cells/100 μm, 13.8 ± 2.2 cells/100 μm, 36.3 ± 6.3 cells/100 μm, and 24.7 ± 2.6 cells/100 μm in the WT, NOD2-KO, WT+BL, and NOD2-KO+BL groups, respectively, at day 10 (Figure 4B). Mice in the WT and NOD2-KO groups had lower apoptosis-positive cells in the cornea, and no significant differences were found between control groups. Apoptotic cell counts in the corneal epithelium were significantly higher in the WT+BL and NOD2-KO+BL groups than in both control groups (all *p* < 0.01). In addition, the NOD2-KO+BL group had lower apoptotic cells in the cornea than the WT+BL group (*p* < 0.01).

### 2.6. Corneal Epithelial Damage

**Figure 5:**
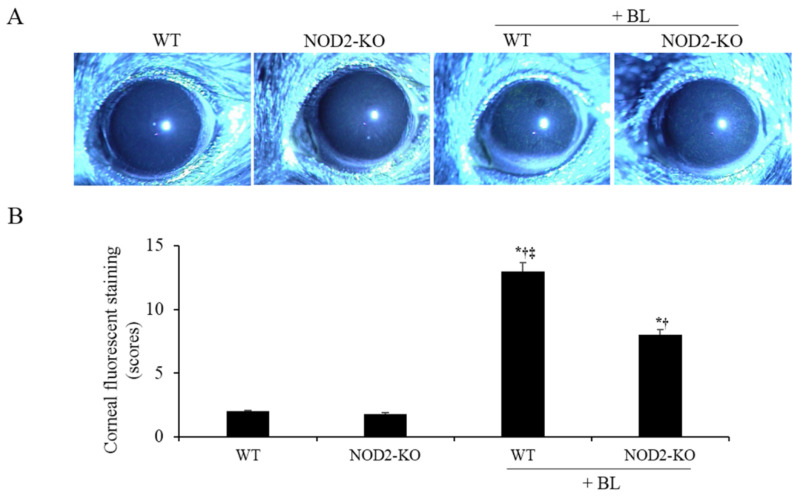
Representative photograph (**A**) and the mean corneal fluorescein staining scores (**B**) in the WT, NOD2-KO, WT+BL, and NOD2-KO+BL groups after 10 days of blue light exposure. * *p* < 0.05 *vs.* WT; ^†^ *p* < 0.05 *vs.* NOD2-KO; ‡ *p* < 0.05 vs. WT + BL WT, wild type; BL, blue light; NOD2-KO, nucleotide-binding oligomerization domain 2 knock-out.

The mean corneal fluorescein staining (CFS) scores were 1.8 ± 0.8 in the WT group, 1.4 ± 0.6 in the NOD2-KO group, 11.2 ± 1.9 in the WT+BL group, and 6.2 ± 1.4 in the NOD2-KO+BL group (Figure 5). After 10 days blue light irritation, both WT and NOD2-KO mice showed higher CFS scores than WT (all *p* < 0.01) and NOD2-KO mice (all *p* < 0.01). However, NOD2-KO mice had significantly lower CFS scores than WT mice (all *p* < 0.01).

## 3. Discussion

Blue light has high photochemical energy and induces cell apoptosis through the overproduction of ROS. Previous studies have demonstrated that excessive ROS can cause severe oxidative stress and deficient autophagy, which in turn can result in retinal pigment epithelial cell damage [5,19,20]. Our previous study has also shown that exposure to blue light can cause corneal epithelial cell death by increasing ROS and inflammatory cytokines, thereby inducing decreased tear volume, reduced tear film break-up time, and increased corneal epithelial damage [7]. However, the ROS-related signaling pathways involved in susceptibility or resistance to blue light-induced cell death have not been investigated in detail.

NOD2 mediates innate immune responses such as inflammation induction, autophagy, or cell death. NOD2 promotes inflammation in Blau-syndrome-associated uveitis [21,22,23]. In dry eye, NOD2 can induce increased production of inflammatory cytokines and T cells on the ocular surface by upregulating RIP2 and NF-κB, resulting in corneal epithelial apoptosis and damage [15,16]. Evidence indicates that NOD2 is directly involved in autophagy and inflammatory responses in Crohn’s disease, and that the increased expression of NOD2 results in NOD2-dependent impaired autophagy responses in dendritic cells [12,17,18].

In the present study, after exposure to blue light, WT and NOD2-KO mice showed higher CFS scores and increased levels of ROS and MDA on the ocular surface than the control mice. In addition, overexpression of NOD2 was observed in the cornea and conjunctiva of WT+BL mice. The results provide evidence that blue light, which causes corneal epithelial damage, can induce ROS and oxidative stress and trigger NOD2 expression on the ocular surface.

Autophagy is generally recognized to play an essential role in homeostasis by mediating the breakdown and conversion of defective organelles within the cells. Recent studies have shown that alterations in autophagy are involved in multiple ocular diseases, such as dry eye, pterygium, and retinal neurodegeneration [6,7,8,19,20]. Furthermore, autophagy plays an important role in blue-light-induced damage. However, the exact changes in autophagy and their underlying regulatory mechanisms are unclear.

In this study, to investigate the relationship between NOD2 and autophagy, the expression of ATG16L1, LC3-II, and p62 on the ocular surface were measured. Upregulated ATG16L1 activation was observed in WT+BL mice after exposure to blue light. In addition, mice in the WT+BL and NOD2-KO+BL groups showed higher expression of LC3-II in the cornea compared to the WT and NOD2-KO groups. However, NOD2-KO+BL mice had a significantly lower expression of p62 and ATG16L1 than WT+BL mice. Our results indicate that NOD2 may be involved in the process of blue-light-induced autophagy responses by regulating ATG16L1 expression. Consistent with our results, NOD2 expression is significantly increased in IBD, and the excessive expression of NOD2 causes impaired autophagy through activation of ATG16L1, thus resulting in cell death in Crohn’s disease [21,22,23].

In conclusion, blue light stimulation can induce impaired autophagy by increasing NOD2 activation on the ocular surface. In addition, the ROS–NOD2–ATG16L1 signaling pathway may be involved in blue-light-induced autophagy responses, resulting in corneal epithelial apoptosis and damage.

## 4. Materials and Methods

### 4.1. Designs of Mouse Model and Experiments

The research protocol was confirmed by the Chonnam National University Medical School Research Institutional Animal Care and Use Committee (no. CNU IACUC-H-2015-11). Maintenance of animals was performed in accordance with the Association for Research in Vision and Ophthalmology statement for the Use of Animals in Ophthalmic and Vision Research. Animals were allowed to acclimate for one week before the experiment began. For the duration of the experiment, mice were kept at 25 °C on a 12-h contrast cycle lighting (bright: 8 a.m.–8 p.m.; dark: 8 p.m.–8 a.m.) under standard laboratory conditions at Chonnam National University Hospital animal facilities.

**Figure 6:**
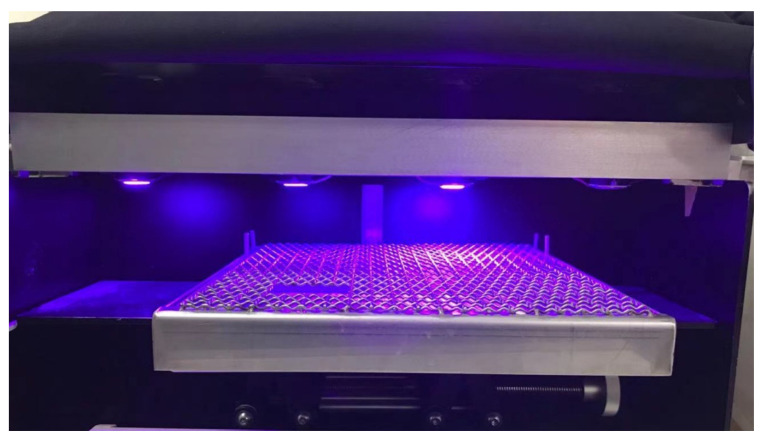
Light emitting diode devices and retaining cages of blue light (410 nm).

Six 8-week female C57BL/six WT and NOD2-KO mice were exposed to blue light (410 nm) at an energy dose of 100 J/cm^2^ twice daily (irradiation began at 9 p.m. and 4 a.m. to avoid variation) for a consecutive 10-day period, as in our previous study [7]. The mice were divided into four groups: WT, NOD2-KO, WT + blue light (WT+BL), and NOD2-KO + blue light (NOD2-KO+BL). The mice in the WT and NOD2-KO groups were not exposed to the blue light, and served as untreated controls during the experiment. Mice were restricted in the cage and the blue light was placed 5 cm above the cage, which vertically stimulated mouse’s head and eye (Figure 6).

At the end of the experiment, the animals were sacrificed. Next, 2′,7′-dichlorodihydrofluorescein diacetate (DCF-DA) for cellular reactive oxygen species (ROS), enzyme-linked immunosorbent assay (ELISA) for malondialdehyde (MDA), immunofluorescent staining for NOD2, and terminal deoxynucleotidyl transferase-mediated dUTP-nick end labeling (TUNEL) staining for apoptosis were performed. In addition, corneal fluorescein staining (CFS) scores were calculated using slit lamp bio-microscopy, while the expression of ATG16L1, light chain 3-II (LC3-II), and p62 were measured using Western blot after 10 days of blue light exposure. Each group consisted of 10 animals, and the experiments were performed on three independent sets of mice.

### 4.2. Measurement of ROS Production in the Cornea and Conjunctiva

ROS levels were evaluated in corneal and conjunctival tissues (6 eyes per group) using a DCF-DA assay. Whole corneal and conjunctival tissues were surgically harvested, cut into pieces, and incubated at 37 °C for 45 min in the presence of 0.5 mg/mL collagenase type D (Roche Applied Science). Following incubation, the samples were crushed to break through a cell strainer with a pore size of 100 μm. The cells were washed with PBS and incubated in the dark with 10 μm DCF-DA (cat. no. D399; Molecular Probes; Thermo Fisher Scientific, Inc) for 30 min at 37 °C. The intensity of fluorescent staining was measured using a FACSCalibur cytometer (BD Biosciences), and the results were shown as the mean percentage increase of DCF-DA fluorescence over the control tissue using Cell Quest software (version 5.2.1; BD Biosciences).

### 4.3. ELISA

Protein levels of lipid peroxidation markers, MDA, were detected using ELISA. The tissues (6 eyes per group) were collected and pooled in lysis buffer containing protease inhibitors for 30 min. Cell extracts were centrifuged at 13,000× *g* for 10 min at 4 °C, and the supernatants were stored at −70 °C until use. Following that, 25 μL of total protein from each sample was pipetted into the assay for MDA (Cell Biolabs, San Diego, CA, USA) using the ELISA kit (catalog no. STA-832, Cell Biolabs, San Diego, CA, USA). The samples were analyzed according to the manufacturer’s instructions [24,25].

### 4.4. Immunofluorescent Staining

Immunofluorescent staining was performed in cryosections of the eye and adnexa, and from each group, one eye was used for staining. Sections were fixed in cold acetone at −20 °C for 6 min and then incubated at room temperature for 45 min with mouse monoclonal anti-NOD2 antibody (1:50; catalog no. ab188646; Santa Cruz, Dallas, TX, USA). Then, the samples were incubated with Alexa Fluor488-conjugated chicken anti-mouse (1:200; catalog no. A21200; Invitrogen, Eugene, OR, USA) for 1 h in the dark at room temperature, followed by three washes in PBS. Sections were then counterstained with 4′,6-diamidino-2-phenylindole (DAPI; catalog no. H-1200; Vector, Burlingame, CA, USA) for 5 min. Digital images of representative areas of the cornea and conjunctiva were captured with a Leica upright microscope (DM2500; Leica Microsystems, Wetzlar, Germany). Cell images were obtained separately with the following fluorescence excitation and emission settings: excitation at 405 and 488 nm and emission between 424 and 472 and 502 and 550 nm for NOD2 expression and DAPI, respectively. The results were expressed by averaging the positive cells from three sections per eye. The mean intensity of staining in each section was measured by analysis software (NIS Elements version 4.1; Nikon, Melville, NY, USA).

### 4.5. Western Blot

Expression of LC3-II and p62 proteins was determined by Western blotting (6 eyes per group). The proteins were extracted from the corneal and conjunctival tissues using a lysis buffer. The lysates were centrifuged at 25,200× *g* for 10 min at 4 °C. The proteins (40 μg) of the samples were loaded by 10% SDS-PAGE for 30 min at 80 V. Following gel loading, the samples were transferred from gel to the membranes and blocked using skim milk (non-fat milk) for 60 min at room temperature. The membranes included rabbit anti-LC3-II (catalog no. ab192890), or rabbit anti-p62 (catalog no. ab56416; primary antibodies obtained from Cell Signaling Technology, Beverly, MA, USA) in 1 × TBST (10 mM Tris-HCl (pH 7.6), 150 mM NaCl, 0.05% Tween-20) overnight at 4 °C. The next day, the membranes were washed three times with 1 × TBST buffer for 5 min and incubated with secondary antibodies in 1 × TBST for 60 min at room temperature. After incubating, the samples were washed three times with TBST for 5 min. The images of immunoreactive bands were captured using an enhanced chemiluminescence system (ECL Blotting Analysis System; Amersham, Arlington Heights, IL, USA). Anti-β-actin was used as an inner control.

### 4.6. Histology

The eye and adnexa were surgically excised, fixed in 4% paraformaldehyde, and embedded in paraffin. Six-micron sections were stained with PAS reagent. Sections obtained from four animals in each group were examined and photographed using a microscope (Olympus Corp., Tokyo, Japan) equipped with a digital camera. Goblet cell density in the superior and inferior conjunctiva was measured in three sections from each eye using image analysis software (Media Cybernetics, Silver Spring, MD, USA) and expressed as the number of goblet cells per 100 μm.

### 4.7. TUNEL Staining

A TUNEL assay was used to detect the 3′ hydroxyl ends of fragmented DNA as an early event in the apoptotic cascade and to identify apoptotic cells. The eye and adnexa were surgically excised, placed in a tube with 4% paraformaldehyde, deparaffinized, washed, rehydrated, fixed, and incubated in 20 μg/mL proteinase K for 10 min at room temperature and then rinsed with PBS for 5 min. The samples were incubated in terminal deoxynucleotidyl transferase, recombinant, enzyme (rTdT) containing equilibration buffer and nucleotide mix for 60 min at 37 °C in the dark, and the reaction was terminated by 2 × saline-sodium citrate (SSC) buffer for 15 min. The samples were washed three times with PBS (5 min per time) and stained using VECTASHIELD^®^ with DAPI. Staining was performed using the DeadEndTM Fluorometric TUNEL System (Promega, Madison, WI, USA) according to the manufacturer’s instructions. The images were observed on a Leica TCS SP5 AOBS laser scanning confocal microscope (Zeiss, Oberkochen, Germany) under LSM 800 10× (N.A. 0.4) oil objective. Cell images were obtained separately with the following fluorescence excitation and emission settings: excitation at 405 and 488 nm and emission between 424–472 nm and 502–550 nm for TUNEL assay and DAPI, respectively. TUNEL positive cells and nuclear staining with DAPI in the cornea were viewed under a fluorescent microscope and expressed as the number of goblet cells per 100 μm.

### 4.8. Ocular Surface Parameters

To investigate corneal epithelial injury, the CFS scores were measured by slit lamp bio-microscopy (magnification, 16×; BQ-900; Haag-Streit, Switzerland) under cobalt blue light. One microliter of 1% sodium fluorescein was instilled into the inferior conjunctival sac of the mouse using a micropipette. Ninety seconds later, punctate staining on the corneal surface was evaluated in a blinded manner. Each cornea was divided into four quadrants and scored, respectively. The CFS scores were calculated using a 4-point scale: 0, absent; 1, slight punctate staining <30 spots; 2, punctate staining >30 spots but not diffuse; 3, severe diffuse staining but no positive plaque; 4, fluorescein-positive plaque. The sum of the four quadrants was regarded as final CFS score, and the maximum score was 16 points [26].

### 4.9. Statistical Analysis

The Statistical Package for Social Sciences (SPSS, version 17.0, Chicago, IL, USA) was used for statistical analyses. Results were presented as mean ± standard deviation (SD). The data were analyzed using one-way analysis of variance (ANOVA) with a Tukey post-hoc analysis; *p* < 0.05 was considered statistically significant.

## Figures and Tables

**Table 1:** ELISA for MDA in the cornea and conjunctiva of WT, NOD2-KO, WT+BL, and NOD2-KO+BL groups, after 10 days of blue light exposure.

	Cornea (pmol/mL)	Conjunctiva (pmol/mL)
WT	7.6 ± 1.3	8.2 ± 2.3
NOD2-KO	8.0 ± 1.6	7.1 ± 1.1
WT+BL	15.0 ± 2.4 *^,†^	14.0 ± 2.5 *^,†^
NOD2-KO+BL	14.2 ± 3.0 *^,†^	11.8 ± 2.2 *^,†^

* *p* < 0.05 vs. WT; ^†^ *p* < 0.05 vs. NOD2-KO. WT, wild type; BL, blue light; NOD2-KO, nucleotide-binding oligomerization domain 2 knock-out.

## Data Availability

The data presented in this study are available on request from the corresponding author.

## References

[1] Moran D.J., Hollows F.C. (1984). Pterygium and ultraviolet radiation: A positive correlation. Br. J. Ophthalmol..

[2] Taylor H.R., West S.K., Rosenthal F.S., Munoz B., Newland H.S., Emmett E.A. (1989). Corneal changes associated with chronic UV irradiation. Arch. Ophthalmol..

[3] Taylor H.R., West S.K., Rosenthal F.S., Munoz B., Newland H.S., Abbey H., Emmett E.A. (1988). Effect of ultraviolet radiation on cataract formation. N. Engl. J. Med..

[4] Cruickshanks K.J., Klein R., Klein B.E. (1993). Sunlight and age-related macular degeneration. The Beaver Dam Eye Study. Arch. Ophthalmol..

[5] Zhang W., Ma Y., Zhang Y., Yang J., He G., Chen S. (2019). Photo-oxidative blue-Light stimulation in retinal pigment epithelium cells promotes exosome secretion and increases the activity of the NLRP3 inflammasome. Curr. Eye. Res..

[6] Hu Z., Zhang Y., Wang J., Mao P., Lv X., Yuan S., Huang Z., Ding Y., Xie P., Liu Q. (2016). Knockout of Ccr2 alleviates photoreceptor cell death in rodent retina exposed to chronic blue light. Cell Death Dis..

[7] Lee H.S., Cui L., Li Y., Choi J.S., Li Z., Kim G.E., Choi W., Yoon K.C. (2016). Influence of light emitting diode-derived blue light overexposure on mouse ocular surface. PLoS ONE.

[8] Peng H., Park J.K., Lavker R.M. (2017). Autophagy and Macropinocytosis: Keeping an Eye on the Corneal/Limbal Epithelia. Investig. Ophthalmol. Vis. Sci..

[9] Barbéa F., Douglas T., Saleh M. (2014). Advances in Nod-like receptors (NLR) biology. Cytokine Growth Factor Rev..

[10] Akira S., Takeda K., Kaisho T. (2001). Toll-like receptors: Critical proteins linking innate and acquired immunity. Nat. Immunol..

[11] Brandstetter C., Mohr L.K.M., Latz E., Holz F.G., Krohne T.U. (2015). Light induces NLRP3 inflammasome activation in retinal pigment epithelial cells via lipofuscin-mediated photooxidative damage. J. Mol. Med. (Berl.).

[12] Cooney R., Baker J., Brain O., Danis B., Pichulik T., Allan P., Ferguson D.J., Campbell B.J., Jewell D., Simmons A. (2010). NOD2 stimulation induces autophagy in dendritic cells influencing bacterial handling and antigen presentation. Nat. Med..

[13] Homer C.R., Richmond A.L., Rebert N.A., Achkar J.P., McDonaid C. (2010). ATG16L1 and NOD2 interact in an autophagy-dependent antibacterial pathway implicated in Crohn’s disease pathogenesis. Gastroenterology.

[14] Saxena A., Lopes F., Poon K.K.H., McKay D.M. (2017). Absence of the NOD2 protein renders epithelia more susceptible to barrier dysfunction due to mitochondrial dysfunction. Am. J. Physiol. Gastrointest. Liver Physiol..

[15] Kim Y.H., Li Z., Cui L., Li Y., Yoon H.J., Choi W., Lee J.B., Liu Z., Yoon K.C. (2019). Expression of nod-like receptors and clinical correlations in patients with dry eye disease. Am. J. Ophthalmol..

[16] Li Y., Jin R., Li L., Yoon H.J., Choi J.H., Park J.H., Liu Z., Li W., Li Z., Yoon K.C. (2019). Expression and role of nucleotide-binding oligomerization domain 2 (NOD2) in the ocular surface of murine dry eye. Investig. Ophthalmol. Vis. Sci..

[17] Schäffler H., Rohde M., Rohde S., Huth A., Gittel N., Hollborn H., Koczan D., Glass Ä., Lamprecht G., Jaster R. (2018). NOD2- and disease-specific gene expression profiles of peripheral blood mononuclear cells from Crohn’s disease patients. World. J. Gastroenterol..

[18] Le Bourhis L., Benko S., Girardin S.E. (2007). Nod1 and Nod2 in innate immunity and human inflammatory disorders. Biochem. Soc. Trans..

[19] Iida T., Yokoyama Y., Wagatsuma K., Hirayama D., Nakase H. (2018). Impact of autophagy of innate immune cells on inflammatory bowel disease. Cells.

[20] Liu Y., Xu H., An M. (2017). mTORC1 regulates apoptosis and cell proliferation in pterygium via targeting autophagy and FGFR3. Sci. Rep..

[21] Strober W., Watanabe T. (2011). NOD2, an intracellular innate immune sensor involved in host defense and Crohn’s disease. Mucosal. Immunol..

[22] Fritz T., Niederreiter L., Adolph T., Blumberg R.S., Kaser A. (2011). Crohn’s disease: NOD2, autophagy and ER stress converge. Gut.

[23] Pillai P., Sobrin L. (2013). Blau syndrome-associated uveitis and the NOD2 gene. Semin. Ophthalmol..

[24] Choi W., Li Z., Oh H.J., Im S.K., Lee S.H., Park S.H., You I.C., Yoon K.C. (2012). Expression of CCR5 and its ligands CCL3, -4, and -5 in the tear film and ocular surface of patients with dry eye disease. Curr. Eye Res..

[25] Park J.W., Li Z., Choi J.S., Oh H.J., Park S.H., Yoon K.C. (2012). Expression of CXCL9, -10, and -11 in the aqueous humor of patients with herpetic endotheliitis. Cornea.

[26] Pauly A., Brignole-Baudouin F., Labbė A., Liang H., Warnet J.M., Baudouin C. (2007). New tools for the evaluation of toxic ocular surface changes in the rat. Investig. Ophthalmol. Vis. Sci..

